# A CRISPR/Cas9 Cleavage System for Capturing Fungal Secondary Metabolite Gene Clusters

**DOI:** 10.4014/jmb.2008.08040

**Published:** 2020-10-30

**Authors:** Xinran Xu, Jin Feng, Peng Zhang, Jie Fan, Wen-Bing Yin

**Affiliations:** 1 State Key Laboratory of Mycology and CAS Key Laboratory of Microbial Physiological and Metabolic Engineering, Institute of Microbiology, Chinese Academy of Sciences, Beijing 000, P.R. China; 2 Savaid Medical School, University of Chinese Academy of Sciences, Beijing 100049, P.R. China

**Keywords:** CRISPR/Cas9, secondary metabolites, heterogeneous expression, biosynthetic gene clusters, filamentous fungi

## Abstract

More and more available fungal genome sequence data reveal a large amount of secondary metabolite (SM) biosynthetic ‘dark matter’ to be discovered. Heterogeneous expression is one of the most effective approaches to exploit these novel natural products, but it is limited by having to clone entire biosynthetic gene clusters (BGCs) without errors. So far, few effective technologies have been developed to manipulate the specific large DNA fragments in filamentous fungi. Here, we developed a fungal BGC-capturing system based on CRISPR/Cas9 cleavage in vitro. In our system, Cas9 protein was purified and CRISPR guide sequences in combination with in vivo yeast assembly were rationally designed. Using targeted cleavages of plasmid DNAs with linear (8.5 kb) or circular (8.5 kb and 28 kb) states, we were able to cleave the plasmids precisely, demonstrating the high efficiency of this system. Furthermore, we successfully captured the entire *Nrc* gene cluster from the genomic DNA of *Neosartorya fischeri*. Our results provide an easy and efficient approach to manipulate fungal genomic DNA based on the in vitro application of Cas9 endonuclease. Our methodology will lay a foundation for capturing entire groups of BGCs in filamentous fungi and accelerate fungal SMs mining.

## Introduction

Fungal secondary metabolites (SMs) exhibit diverse bioactivities and have been applied widely in different fields including industry, agriculture and medicine [1]. In recent years, a vast number of biosynthetic gene clusters (BGCs) have been found by high-throughput genome sequencing [2, 3]. However, most of the gene clusters are transcriptionally inactive, *i.e.* ‘silent’, under standard laboratory or naturally observed cultivation [3-5]. To activate these ‘silent’ BGCs, genome mining strategies either by cloning and expressing the entire group of BGCs in heterologous hosts or manipulating regulators directly in native hosts have been developed for novel metabolite discovery and their biosynthetic analysis [6]. So far, heterologous clusters are commonly expressed in fungi, especially in engineered *Aspergillus* species and *Saccharomyces cerevisiae* [7, 8]. A number of compounds, such as asperfuranone [9], geodin [10], neosartoricin B [11], citreoviridin [12] and aspercryptin [13] have been discovered by heterologous expression.

Even so, in vitro isolation and cloning of giant DNA fragments have remained key technical challenges in molecular biology due to the large size of fungal ‘silent’ BGCs (10 to 130 kb) [14]. To obtain large fragments from native strains, transformation-associated recombination (TAR)-cloning has been utilized to manipulate full-length genes with fragment size up to 250 kb based on in vivo homologous recombination in *S. cerevisiae* [15]. This strategy requires considerable effort and time to screen hundreds of colonies, but leads to low efficiency and specificity [16]. Notably, the TAR-cloning strategy has not been successful with DNA fragment assemblies in fungi. On the other hand, fungal artificial chromosome (FAC) systems were reported to shuttle and stably maintain large DNA fragments (>150 kb) in both *Escherichia coli* and filamentous fungi. The method utilizes unbiased random shear bacterial artificial chromosome (BAC) technology coupled with an autonomous fungal replicating element AMA1, and miscellaneous selection toward target fragments [14, 17]. However, it is laborious due to the amounts of colony screening and also costs a lot for colony sequencing.

Instead, the clustered regularly interspaced short palindromic repeats-associated protein (CRISPR/Cas) system from *Streptococcus pyogenes* has been used in numerous model organisms recently to edit the genome with high efficiency, versatility and specificity in vivo [18, 19]. Firstly, an effector complex is assembled between the Cas9 system with mature single guide RNA (sgRNA), which comprises CRISPR RNA (crRNA) and trans-activating crRNA (tracrRNA)-derived sequences. Afterwards, this Cas9-sgRNA complex recognizes target DNA guided by 5’ sgRNA and specifically cleaves double-stranded DNA (dsDNA) to complete the editing process [20]. Notably, TAR cloning or Gibson was combined with a CRISPR/Cas9 system for isolation and cloning of large genomic sequences from human or bacteria more efficiently and specifically [16, 21, 22]. However, no methods have been reported yet to specifically capture an entire BGC in filamentous fungal genomes.

In this study, we present a CRISPR/Cas9 system in vitro for cleavage of fungal BGCs. The feasibility and efficiency of this system will be verified by cleaving plasmids with different shapes and sizes. Subsequently, the genomic DNA extracted from fungi will be cleaved by RNA-guided Cas9 endonuclease in vitro. The target gene clusters with large size will be inserted into vectors for heterogeneous expression. Compared to currently available methods, our method is a rapid approach to obtain target gene clusters accurately in filamentous fungal genomes.

## Materials and Methods 

### Strains and Plasmids

*E. coli* Rosetta 2 (DE3) and *E. coli* EPI300 were purchased from Merck Millipore and Epicentre, respectively. *S. cerevisiae* BJ5464-NpgA (*MATα ura3-52 his3-Δ200 leu2-Δ1 trp1 pep4::HIS3 prb1Δ1.6R can1 GAL*) was used as the host for homologous recombination. Fungal strains used in this study are *Neosartorya fischeri* and *Pestalotiopsis fici*. Plasmids are listed in Table 1 and oligonucleotide sequences are shown in Table S1. pX330 vector [23, 24] was used to obtain gene sequence of Cas9 and used as target cleavage plasmid. pET-28a(+) vector was used to produce Cas9 protein. pCC1BAC vector was the starting vector of shuttle plasmids constructed in this study. Another target plasmid pYPZ37 (28.0 kb) harboring *Pfma* gene cluster was constructed in the previous study [25].

### Expression and Purification of Cas9

The Cas9-expressing plasmid pYJF22 was generated as follows: a 4,104 bp-fragment containing Cas9 open reading frame (ORF) was obtained by PCR from pX330 with designed primers (Table S1) and inserted into the pET-28a(+) vector according to the ligation and cloning method to give the plasmid of pYJF22. The construct was then introduced into *E. coli* Rosetta 2 (DE3). The recombinant protein was purified as described previously [26].

### Synthesis and Purification of sgRNA 

CRSIPR design tool (http://crispr.mit.edu/) was used to identify CRISPR guide sequences. A double-stranded transcription template was prepared by amplifying a single-stranded oligonucleotide by PCR amplification of antisense template oligonucleotide with the T7 promoter sequence as a forward primer and the 3′ end of the antisense template as a reverse primer. Primers used for synthesis of sgRNA are sgRNA-F/R listed in Table S1. PCR reaction components are as follows: 4 μl of 2.5 mM dNTPs, 5 μl 5× Q5 reaction buffer, 0.5 μl Q5 High-fidelity DNA polymerase (2 U/μl), 2.5 μl target-specific primer X-sgRNA-P (10 μM), 1 μl of universal primer sgRNA-F (10 μM) and 2.5 μl of universal primer sgRNA-R(10 μM) without any template as well as sterile water to a total volume of 25 μl. This reaction was performed as follows: heat the reaction to 98°C for 30 second (sec) to denature, then perform 30 cycles of PCR [98°C for 10 sec (denaturation), 55°C for 20 sec (annealing) and 72°C for 15 sec (elongation)] followed by ﬁnal elongation at 72°C for 2 min [27].

The synthesized DNA mentioned above was purified using DNA Clean & Concentrator Kits (Zymo Research, USA). A concentration of 30-100 ng/μl purified DNA with A_260/280_ > 1.8 was measured by NanoDrop. Subsequently, in vitro transcription was performed using a T7 High Efficiency Transcription Kit (Transgen). After transcription, large quantities of RNA were purified by EasyPure RNA Purification Kit (Transgen) to give sgRNA. A concentration of 300-1,000 ng/μl synthesized sgRNA with A_260/280_ > 2.0 was measured for purity detection by NanoDrop. Finally, synthesized sgRNA was diluted to a concentration of 300 -1,000 nM with RNase-free water for the preparation of Cas9 cleavage assays.

### In Vitro Cas9 Endonuclease Cleavage Assays for Plasmids pX330 and pYPZ37

Cas9-sgRNA complexes were constituted before cleavage by incubating Cas9 endonuclease with in vitro transcribed sgRNA in the reaction mixture containing 5× cleavage buffer (100 mM HEPES [pH 7.5], 500 mM KCl, 25% glycerol, 5 mM DTT, 2.5 mM EDTA [pH 8.0], 10 mM MgCl_2_) and nuclease-free H_2_O. After incubation at 30°C for 20 min, the cleavage reaction was initiated by adding the target DNA to the reaction mixture immediately at 37°C. For the cleavage of linearized pX330, 400 ng of DNA, pX330-sgRNA (30 nM) and Cas9 Nuclease (30 nM) were added in the cleavage reaction system to a total reaction volume of 30 μl. The cleavage reaction was carried out at 37°C for 2 h and overnight, respectively. The cleavage efficiency and off-target effects were assessed using pulsed-field gel electrophoresis (PFGE).

For the cleavage of circular pX330, 400 ng of circular pX330 DNA, different amounts of Cas9 and sgRNA (mole ratio 1:1) at final concentration (15, 30, 60, and 120 nM) were added into the reaction mixture to a total reaction volume of 30 μl. After incubation, the samples were used to assess the cleavage efficiency and off-target effects using agarose gel electrophoresis.

For the cleavage of HMW plasmid pYPZ37, pYPZ37-sgRNA1 (30 nM), pYPZ37-sgRNA2 (30 nM) and Cas9 Nuclease (60 nM) and different amounts of DNA at final concentration (50, 100, 200, 500, and 1,000 ng) were added into the reaction mixture to a total reaction volume of 50 μl. The cleavage efficiency and off-target effects were assessed using PFGE.

The PFGE was performed with Lambda DNA-Mono Cut Mix as marker in 1% Megabase agarose gel in 0.5× TBE buffer with the Bio-Rad CHEF Mapper XA System by loading the gel with agarose plugs (or 10 μl purified plasmids). The CHEF Mapper XA System was set to auto-algorithm program with 5-250 kb parameters (6 V/cm, 0.22-21.79 s, 15 h 16 min, 120°, circulation at 14°C), and the gel was strained by GoldView for visualization using standard gel imaging systems.

### Preparation of Fungal DNA

In this study, fungal strains *N. fischeri* and *P. fici* were cultivated at 25°C on potato dextrose agar medium (PDA) for 3 days. For the cleavage reaction in vitro and in vivo, fungal genomic DNA was extracted from lyophilized mycelia by phenol: chloroform: isoamyl alcohol (25:24:1, pH > 7.8) as described previously [28].

### Construction of Shuttle pCC1BAC Vectors

The *pyrG* and *ribo* auxotrophy markers were amplified from *A. fumigatus* genomic DNA by using primer pairs Nrc-PyrG-F/R and riboWA-F/R, while the 5’ and 3’ regions of the *wA* gene were amplified from *A. nidulans* genomic DNA by using primers wAup-Nrc-F/R. All three DNA fragments for in vivo yeast recombination contained a minimum of 35 bp overlapping bases with the flanking fragments. The terminal sequence overlaps (up to 1kb) of the target gene clusters (*Nrc* and *Pfma*) were prepared by PCR amplification. The PCR primers consisted of an ~25 bp sequence that annealed to the vector template and an ~25 bp overhang that overlapped with one end of the target gene cluster. Then these terminal sequence overlaps were ligated to shuttle pCC1BAC vectors by the yeast homologous recombination method to obtain constructs pYJF27and pYJF28. For in vivo Cas9 cleavage application, these two constructs were linearized by NheI and BamHI for further transformation.

### In Vitro Cas9 Endonuclease Cleavage for Fungal Genomic DNA

The reactions were performed as in vitro Cas9 endonuclease cleavage assays for plasmids. For the cleavage of *N. fischeri* gDNA, 400ng gDNA, Nrc-sgRNA1 (30 nM), Nrc-sgRNA2 (30 nM) and Cas9 endonuclease (60 nM) were added into the cleavage reaction system to a total reaction volume of 100 μl. For the cleavage of *P. fici* gDNA, 7101-sgRNA1 (30 nM), 7101-sgRNA2 (30 nM), Cas9 endonuclease (60 nM) and different amounts of DNA at final concentrations (1, 2, 5, 10, and 20 μg) were added into the reaction assay to a total reaction volume of 100 μl. The obtained DNA was concentrated by ethanol precipitation method [27].

Afterwards, all of the gDNA cleaved by Cas9, 1 μg of the linearized shuttle pYJF27 or pYJF28, 200 μl suspension of freshly prepared yeast (BJ5464-NpgA) competent cells (~10^8^) and 600 μl solution III (Zymo Research, USA) were added into the assembled reaction. The homologous recombination in yeast was performed as the standard manuscript (Zymo Research). Further colonies were verified by extracting their plasmids from SDCt media and subsequent PCR by using diagnostic primers *Nrc* F5/R5 (Table S1). Plasmid isolation from yeast transformants was performed by using a Zymoprep Kit (Yeast Plasmid Miniprep I, Zymo Research). Plasmids with more amount were further extracted from *E. coli* transformants by PlasmidMini Kit I (Omega, USA) and verified by restriction enzyme digestion (KpnI and NcoI).

### Preparation of High-Molecular-Weight (HMW) DNA

*P. fici* protoplasts were prepared as described previously [29]. According to previous studies [14] on preparing low melting agarose plugs of HMW DNA, the pellet protoplasts (~5×10^8^) were resuspended with the HMW DNA preparation buffer to a total volume of 0.6 ml. An equal volume of 1% low melting agarose was then added to the buffer and incubated at 45°C. This was sufficient to make 10 plugs (about 100 μl per plug) which solidified at 4°C. The plugs were then incubated in 1 ml of lysis buffer/plug (0.5 M EDTA (pH 9.0), 1% lauryl sarcosine, 1 mg/ml proteinase K) at 50°C for 48 h. Finally, the plugs were extensively washed in 10-20 volumes of the following buffers for one hour in each wash: once with buffer 1 (0.5 M EDTA [pH 9.0-9.3] at 50°C), once with buffer 2 (0.05 M EDTA [pH 8.0] on ice), three times with buffer 3 (ice-cold TE plus 0.1 mM phenylmethyl sulfonyl fluoride (PMSF) on ice), three times with buffer 4 (ice-cold TE on ice) and finally all plugs were stored in TE buffer at 4°C.

### In-Gel Cas9 Digestion

In-gel Cas9 digestion was performed as described previously [27]. For the in-gel Cas9 cleavage of each target sequence, six agarose blocks were used. Additional wash with 0.1× wash buffer (diluted from 1× wash buffer with ultrapure water) was immediately performed before Cas9 digestion for diluting the EDTA. After discarding the wash buffer, 100 μl of 1× cleavage buffer (diluted from 5× cleavage buffer) was added into each tube and the agarose blocks equilibrated at room temperature for 30 min. During the equilibration, the assay of Cas9 with sgRNAs was assembled by mixing 60 μl of 5× cleavage buffer, 15 μl of DTT (10 mM), 30 μl of Pfma-sgRNA1 (300 ng/μl), 30 μl of Pfma-sgRNA2 (300 ng/μl), 3 μl of Cas9 (20 μM) and RNase-free water to a total volume of 300 μl and incubated at 37°C for 20 min. Subsequently, 50 μl of the prepared cleavage solution was added into each micro centrifuge tube (the 1× cleavage buffer was discarded) and incubated at 37°C for 2 h allowing the diffusion and digestion by Cas9 to occur in the agarose matrix. Then, the cleavage solution was discarded and all of the available blocks for each target sequence were transferred into a clean 1.5 ml micro centrifuge tube. The plugs were washed with 1 ml of 0.1× wash buffer and then suspended in 500 μl of 1× GELase buffer (diluted from 50× GELase buffer with ultrapure water) for 1 h incubation at room temperature. After discarding the 1× GELase buffer, the agarose blocks were thoroughly melted by incubating them in a 70°C water bath for 5 min. The consistency and appearance of the agarose were assessed after gently flicking the micro centrifuge tube. When the sample has turned into liquid, it can be transferred into a micro centrifuge tube containing the molten agarose into a 45°C water bath and equilibrated for 10 min. Finally, 1 μl of GELase enzyme was slowly added to the agarose and stirred with a 10 μl tip very gently to avoid shearing of the long DNA fragments for further incubation at 45°C for 30 min.

After GELase digestion, the sample was cooled to room temperature for 5 min and flicked gently to examine the appearance of the agarose. If the sample remains liquid, an equal volume (~170 μl) of 5 M CH_3_COO(NH_4_)_2_ was added to the digested agarose and gently inverted several times to mix thoroughly. Then, 4× volume (~680 μl) of room-temperature ethanol was added to the micro centrifuge tube and gently inverted several times to mix thoroughly. After incubation at room temperature for 2 h, the DNA was collected as pellet by centrifugation at 12,000 g at room temperature for 15 min. After washing with 70% ethanol twice, 20 μl of ultrapure water was added into the pellet.

## Results

### Design of In Vitro CRISPR/Cas9 Cleavage System

**Fig. 1:**
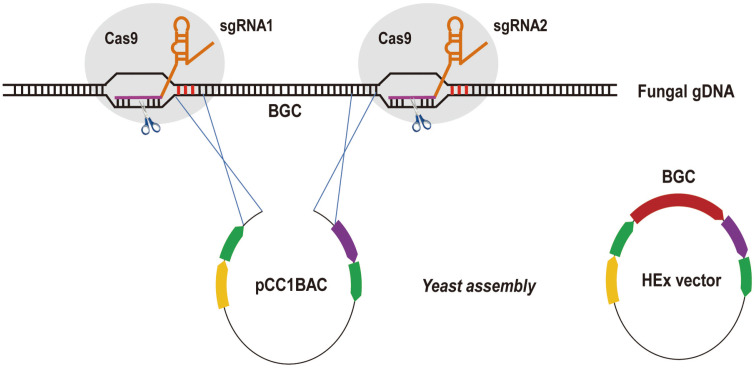
Outline of in vitro CRISPR/Cas9 cleavage system capturing entire gene clusters in fungi. The genomic DNA extracted from fungi could be cleaved by RNA-guided Cas9 nuclease in vitro, and the target gene clusters are directly linked to heterologous expression vectors.

To build the CRISPR/Cas9 system in vitro for cleaving the fungal BGCs (Fig. 1), we first introduced the coding sequence of Cas9 endonuclease into *E. coli* for protein overproduction. Based on the information provided by the codon usage database (http://www.kazusa.or.jp/codon/), the ORF of Cas9 gene was amplified from the vector pX330 and subsequently ligated to protein expression vector pET-28a(+) to give pYJF22 (Fig. S1A in the Supplementary Information (SI)). The recombinant His6-tagged protein was purified to near homogeneity as confirmed on SDS-PAGE, revealing that its molecular weight was consistent with the predicted value of 164.5 kDa (Fig. S1B).

The CRSIPR design tool (http://crispr.mit.edu/) was used to identify CRISPR guide sequences within the two 1 kb regions beginning with the 250 bp nearest the hooks (Fig. S2A). As an in vitro transcription based on the T7 RNA polymerase was later used, the CRISPR guide sequence had to conform to the rules governing efficient and precise initiation of the T7 promoter. In detail, the CRISPR guide sequence must start with a guanine (G), followed by a purine (G/A) with ‘G’ being significantly more preferred and finally an optional third ‘G’. Thus, the CRISPR guide sequence should start with ‘5-GGN’, implying the difficulty to obtain a CRISPR guide sequence. Thus, one additional ‘G’ was added to the 5′ end of a 20 bp CRISPR guide sequence according to a previous strategy by Jiang [21]. As mentioned above, an RNA-guided CRISPR/Cas9 system was designed to give several groups of Cas9-sgRNA for further cleavage application.

### Target Cleavage of Linearized or Circular dsDNA by CRISPR/Cas9 

**Fig. 2:**
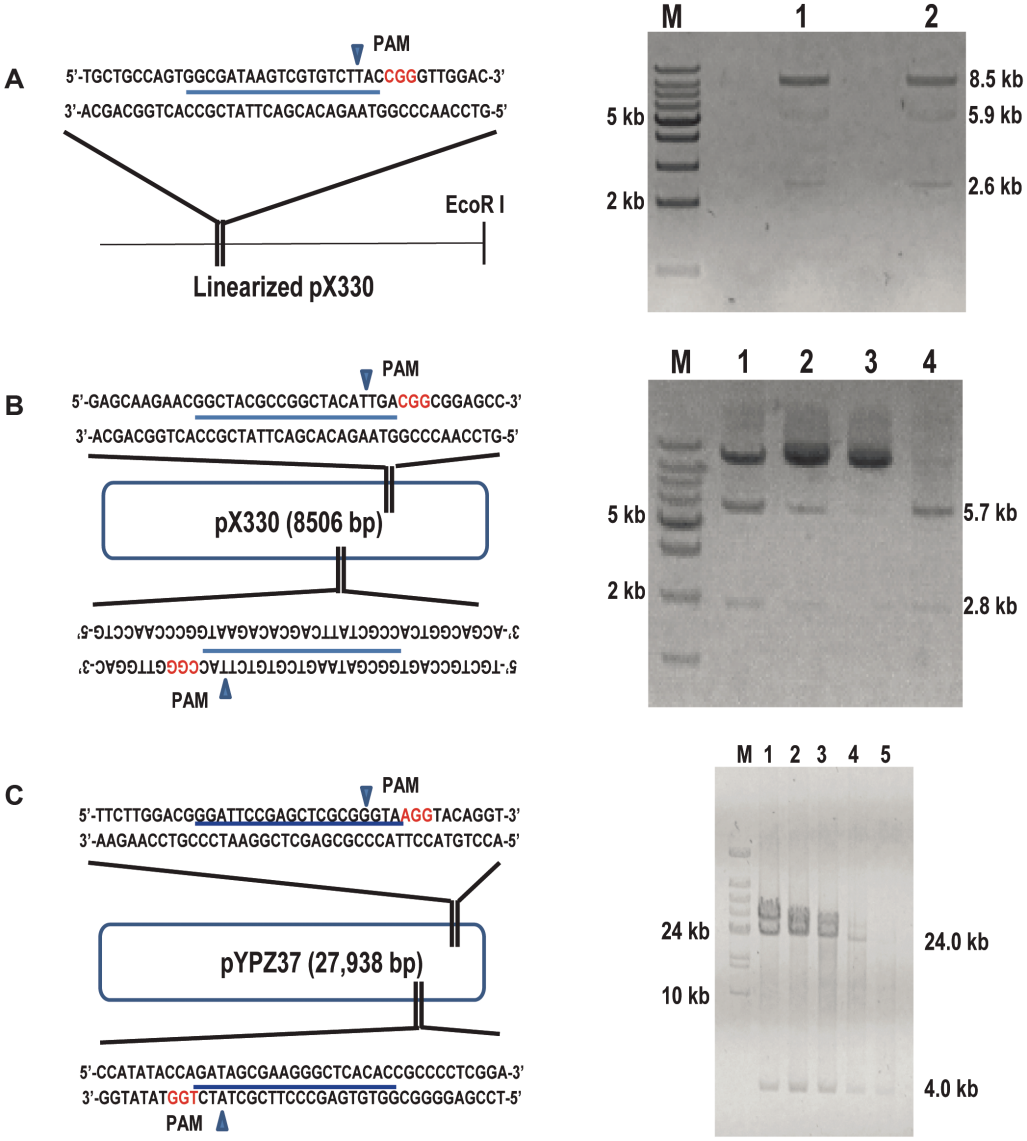
Cleavage of linearized or circular dsDNA by CRISPR/Cas9 in vitro. (**A**) Designed sgRNA and linearized pX330 by EcoRI (200 ng/μl, 8.5 kb) cleaved by Cas9 endonuclease. Lane M: marker; Lanes 1 and 2: linearized pX330 cleaved by Cas9 at 37°C for 2 h and overnight, respectively. (**B**) Designed sgRNAs and circular pX330 (200 ng/μl, 8.5 kb) cleaved by Cas9 endonuclease. Lane M: marker; Lanes 1-4: circular pX330 cleaved by Cas9 and sgRNA at different concentrations of 60 nM, 30 nM, 15 nM and 120 nM. (**C**) Designed sgRNAs and circular pYPZ37 (415 ng/μl, 28.0 kb) cleaved by Cas9 endonuclease. Lane M: marker; Lane 1-5: circular pYPZ37 cleaved by Cas9 at different amounts of 1,000 ng, 500 ng, 200 ng, 100 ng, and 50 ng.

As a proof-of-principle, we proceeded to apply RNA-guided Cas9 endonuclease in the following cleavage of linearized or circular dsDNA in vitro based on the principle of the CRISPR/Cas9 system. The effector complex, termed Cas9-sgRNA complex, was constituted before cleavage by incubating Cas9 endonuclease with the in vitro transcribed sgRNA. Referring to previous study, it is essential to keep the molar ratio of Cas9 and sgRNA per target site at 10:10:1 or higher to obtain the best cleavage efficiency [30]. Subsequently, one protospacer (S1) on the linearized plasmid pX330 by EcoRI was selected to transcribe pX330-sgRNA1. When pX330-sgRNA1 was complexed with Cas9, the linearized pX330 (8.5 kb) was cleaved at the predicted position to generate two dsDNA fragments whose lengths were 5.9 kb and 2.6 kb, respectively (Fig. 2A). The gel electrophoresis analysis of pX330 cleavage samples indicated that overnight digestion was more efficient, although the linearized plasmid pX330 had been cut in two hours. Therefore, the overnight incubation time was used in the following assays.

To investigate the cleavage reaction of two sgRNAs complexed with Cas9, two protospacers (S1 and S2) on pX330 were selected to transcribe pX330-sgRNA1 and pX330-sgRNA2. Incubation of the circular pX330 (8.5 kb) DNA with Cas9 endonuclease, pX330-sgRNA1 as well as pX330-sgRNA2 led to the cleavage at the predicted position, yielding two fragments with sizes of 5.7 kb and 2.8 kb (Fig. 2B). Further incubation of Cas9 with sgRNAs (mole ratio 1:1) was carried out at different final concentrations (15, 30, 60 and 120 nM). Clear bands at the expected lengths were observed in each of the four lanes, but with higher digestion efficiency at higher concentration. Surprisingly, the circular pX330 was completely cleaved into dsDNA fragments at the final concentration of 120 nM. Thus, a high final concentration of Cas9 and sgRNA is essential for the CRISPR/Cas9 in vitro cleavage system.

To investigate the cleavage efficiency for dsDNA with high molecular weight (HMW) by CRISPR/Cas9, we proceeded with the cleavage assay of the circular plasmid pYPZ37 (28.0 kb) [25]. In analogy to the incubation of pX330-sgRNAs, two protospacers (S1 and S2) on pYPZ37 were selected to transcribe pYPZ37-sgRNA1 and pYPZ37-sgRNA2. When pYPZ37-sgRNA1 and pYPZ37-sgRNA2 were complexed with Cas9, the circular pYPZ37 was cleaved at the predicted position to generate two fragments with sizes of 24.0 kb and 4.0 kb, respectively (Fig. 2C). Incubation with different amounts of DNA (50, 100, 200, 500, and 1,000 ng) were further carried out, indicating more cleaved product obtained with higher amount of DNA by PFGE. This proved the sufficient cleavage efficiency of the RNA-guided Cas9 system in vitro, even for high amounts of DNA with high molecular weight. Taken together, the in vitro CRISPR/Cas9 system can accurately cut linear or circular dsDNA, and the final concentrations of dsDNA, Cas9 an d sgRNA will affect the cleavage efficiencies.

### Capturing Entire Fungal BGCs with CRISPR/Cas9 System

**Fig. 3:**
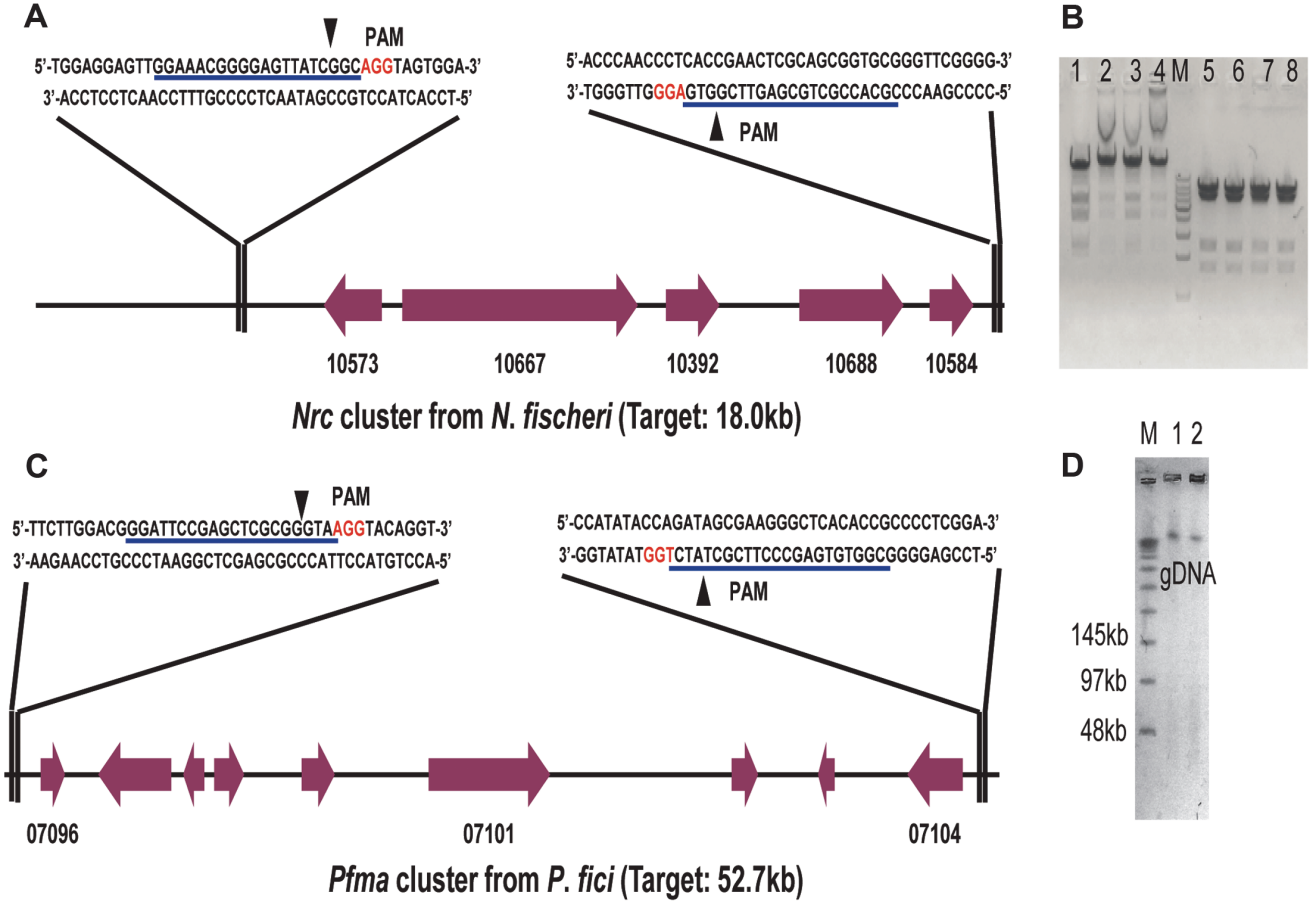
Capturing the fungal gene cluster with CRISPR/Cas9 cleavage system. (**A**) SgRNA pairs designed to target *Nrc* gene cluster from *N. fischeri*. (**B**) Verification of the obtained construct pYJF31 containing *Nrc* gene cluster by restriction endonuclease digestion. Lane M: marker; Lanes 1-4: plasmids digested by KpnI. Lengths of target fragments are 15,631 bp, 6,209 bp, 4,432 bp, 2,782 bp and 2,388 bp; Lane 5-8: plasmids digested by NcoI. Lengths of target fragments are 7,600 bp, 7,348 bp, 6,271 bp, 5,975 bp, 2,511 bp and 1,737 bp. (**C**) SgRNA pairs designed to target genome *Pfma* gene cluster from *P. fici*. (**D**) Genomic DNA of *P. fici* analyzed using PFGE. Lane M: marker; Lane 1: *P. fici* gDNA without Cas9 cleavage; Lane 2: *P. fici* gDNA cleaved by Cas9 in low melting agarose plugs.

Having identified the in vitro CRISPR/Cas9 cleavage system, it was then applied to manipulate the fungal genomic DNA. Two gene clusters, named *Nrc* and *Pfma*, with different lengths were selected to be edited. *Nrc* gene cluster with a length of 18.0 kb derived from *N. fischeri* (Fig. 3A) is responsible for the synthesis of neosartoricin B with immunomodulatory activity [11]. *Pfma* is a DHN melanin biosynthetic gene cluster in *P. fici* with a length of 52.7 kb (Fig. 3C) [25]. At first, we constructed *E. coli*-*yeast*-*Aspergillus* shuttle pCC1BAC vectors by using homologous recombination in *S. cerevisiae*, which enables insertion of fragments with sizes up to a couple of 100 kb (Figs. 1 and S3). As a result, the acquired constructs pCC1BAC-wA-pyrG-Nrc (pYJF27) and pCC1BAC-wA-ribo-Pfma (pYJF28) consisted of (1) the linearized pCC1BAC vector, (2) yeast centromere sequence (CEN), autonomously replicating sequence (ARS), and a URA3 marker, (3) a 5’ *wA* -3’ *wA* cassette synthesized by fusion PCR, (4) the *pyrG* and *ribo* auxotrophy markers and (5) the terminal sequence overlaps (~1000 nucleotides) of the target gene clusters.

Subsequently, two sgRNAs necessary for cleaving gene clusters were synthesized in vitro and combined with Cas9-treated fungal genomic DNA. With linearized pYJF27 and pYJF28 by NheI and BamHI, respectively, in vivo recombination in *S. cerevisiae* between the hooks and Cas9-treated genomic DNAs containing *Nrc* or *Pfma* gene cluster was carried out. After 2-3 days of cultivation, two positive colonies harboring a 31.4 kb fragment were obtained from the mixture containing 1 mg of *N. fischeri* genomic DNA and 1 mg of linearized pYJF27 plasmid by PCR using the diagnostic primer pair *Nrc* F5/R5 and the following restriction enzyme digestions. Thus, we successfully captured the entire BGC of *Nrc* by RNA-guided Cas9 system in *N. fischeri* (Fig. 3B).

## Discussion

In the post-genomic era, the heterologous expression approach has become one of the most effective ways to activate ‘silent’ gene clusters for mining fungal SM “dark matter” [31, 32]. Usually, a fungal genome includes about 20-90 BGCs which span 10-130 kb for each BGC [1, 17]. Therefore, a bottleneck for successful heterologous expression of a BGC is the efficient cloning of large DNA fragments. Currently, available techniques for large DNA fragment cloning are mostly based on PCR or restriction-enzyme-based methods [33], which are severely constrained by errors, restriction site limitation and low efﬁciency when a gene cluster spans dozens of kilobases [20]. Though TAR had been developed for the efficient assembly of BGCs from human [15] and bacteria [34], it is still challenging to clone large DNA fragments from fungi. In a pioneer group, Keller lab developed a fungal artificial chromosome (FAC) system that can capture a large DNA fragment (over 100 kb). However, this technique is high cost and laborious because it is based on the screening and sequencing of large-scale colonies [14, 17]. Another limitation is that FAC DNA fragments are randomly selected and cannot define the exact border of a BGC. Recently, the Cas9 as a programmable endonuclease has demonstrated its advantages in precisely editing genomic DNA [35-36]. Based on the CRISPR/Cas9 technology, several in vivo genetic manipulation methods have been established in filamentous fungi [24,37-39]. However, the application of the CRISPR/Cas9 system in vitro for capturing/editing an entire BGC is limited.

To capture entire BGCs from fungi, we conducted many trials using methods including TAR, and SOE (splicing by overlap extension)-PCR based yeast recombination [40] but failed in the past years. Considering the differences between eukaryotic and prokaryotic cells, we presume that some difficulties are not unknown for manipulating eukaryotic genomic DNA. The advantages of the CRISPR/Cas9 system in gene editing encouraged us to achieve the entire BGCs capturing. When designing the CRISPR/Cas9 system, we first tried to use the CRISPR/Cas9 system to cleave linearized and circular dsDNA in vitro. Among the many possible reasons, a variety of sgRNAs were applied to the CRISPR/Cas9 system, and the cleavage and cloning results suggested that the selection of sgRNAs did not appear to affect the cleavage and cloning efﬁciency [21]. Using two plasmid DNAs with different sizes of pX330 (8.5 kb) and pYPZ37 (28.0 kb) as samples, we found that the two plasmids were digested efficiently as expected (Figs. 2A and 2B). Our CRISPR/Cas9 system revealed sufficient cleavage in vitro, indicating that it can be excluded for restricting the efficiency of the cleavage CRISPR/Cas9 system. The success of the trial in plasmid cleavage encourages us for our next capture of the entire group of BGCs from fungal genomic DNA. The size of the *Nrc* gene cluster is about 18.0 kb from *N. fischeri*, with the genome size of 32Mb [41]. By the extraction of genomic DNA in combination with yeast recombination, the entire gene cluster *Nrc* was target isolated and constructed onto BAC vectors (Figs. 3A and 3B).

To further utilize the in vitro CRISPR/Cas9 cleavage system for larger cluster editing, we attempted to prepare HMW DNA by low melting agarose plugs [14] and in-gel Cas9 digestion [27]. This will avoid the destruction of fungal genomic DNA by phenol: chloroform: isoamyl alcohol. Therefore, the CRISPR/Cas9 cleavage system was extended for editing the fungal strain *P. fici* with the help of PEG-mediated protoplast transformation [29]. The digestion of protoplast was performed according to the preparation of HMW DNA in *A. terreus* [14]. Subsequently, these treated protoplasts were digested by Cas9-sgRNA complexes and used to assess the cleavage efficiency by PFGE. Although we obtained a relatively high-quality genomic DNA of *P. fici* (Fig. 3D), it is still difficult to isolate the *Pfma* gene cluster from *P. fici* genome. Correspondingly, the length of the *Pfma* gene cluster was one thousandth of the total gene length of *P. fici* genome DNA at 52 Mb [42]. The ratio between the target gene cluster and the fungal genome is a key possible reason for obtaining the target gene cluster. Although we failed to obtain a gene cluster greater than 50 kb, it should be possible to extend the method to clusters larger than this. Therefore, the entire BGC capture efﬁciency could be improved by procedure optimization like desalting after end repair, using super competent cells and applying electro transformation approaches.

In conclusion, we developed an in vitro CRISPR/Cas9 cleavage system for capturing the target gene clusters from filamentous fungi. Using this system, the linear or circular DNA can be cleaved efficiently. It is a possible replacement for the restriction enzymes due to having no limitation of site/place selection. Ultimately, we captured the entire gene cluster of *Nrc* from *N. fischeri*. This is a big step for obtaining BGCs precisely without PCR amplification steps from filamentous fungi. Our results provide a new approach to manipulate fungal genomic DNA and an efficient tool for mining fungal BGCs for heterologous expression.

## Supplemental Material

Supplementary data for this paper are available on-line only at http://jmb.or.kr.

## Figures and Tables

**Table 1:** Plasmids used in this study.

Plasmid name	Description	References
pX330	*Streptococcus pyogenes* *Cas9*, *Amp*	[23-24]
pYPZ37	*URA3*, *AfRibo*, *wA flanking*, *Amp*, *Pfma* *cluster* (*PfmaA-E*, *PfmaG*, *gpdA(p)::PfmaH*)	[25]
pET-28a	Protein expression vector	Invitrogen
pCC1BAC	BAC vector	[21]
pYJF22	*S. pyogenes Cas9*, *Kan*	This study
pYJF24	URA3, PyrG, Cm, wA flanking in pCC1BAC-	This study
pYJF26	URA3, AfRibo, Cm, wA flanking in pCC1BAC	This study
pYJF27	Nrc-5F and 3F in pYJF24	This study
pYJF28	7101-5F and 3F in pYJF26	This study
pYJF31	Nrc-Cluster in pYJF27	This study
