# Supplementary material for: A CRISPR/Cas9 Cleavage System for Capturing Fungal Secondary Metabolite Gene Clusters
Source: J Microbiol Biotechnol. 2020 Oct 30;31(1):8–15. doi: 10.4014/jmb.2008.08040 (PMC9705949; doi:10.4014/jmb.2008.08040)
Supplement: Supplementary file 1 [file jmb-31-1-8-supple.pdf]

https://doi.org/10.4014/jmb.2008.08040

Supplemental Figures

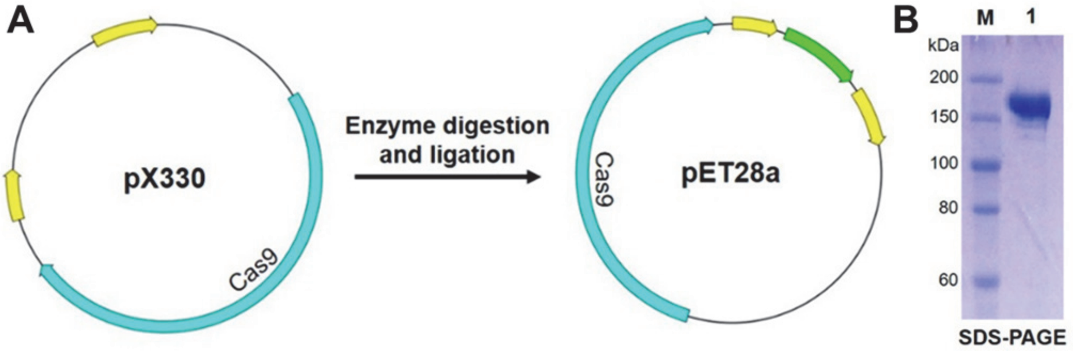

Figure S1. Protein expression and purification of Cas9 endonuclease. (A) Construction of expression plasmid pYJF22 harboring pET-28a(+)-Cas9. (B) The purified recombinant histidine-tagged Cas9 were separated on a 12% SDS-PAGE. Lane M: marker; Lane 1: Cas9 (Predicted molecular weight: 164.5 kDa).

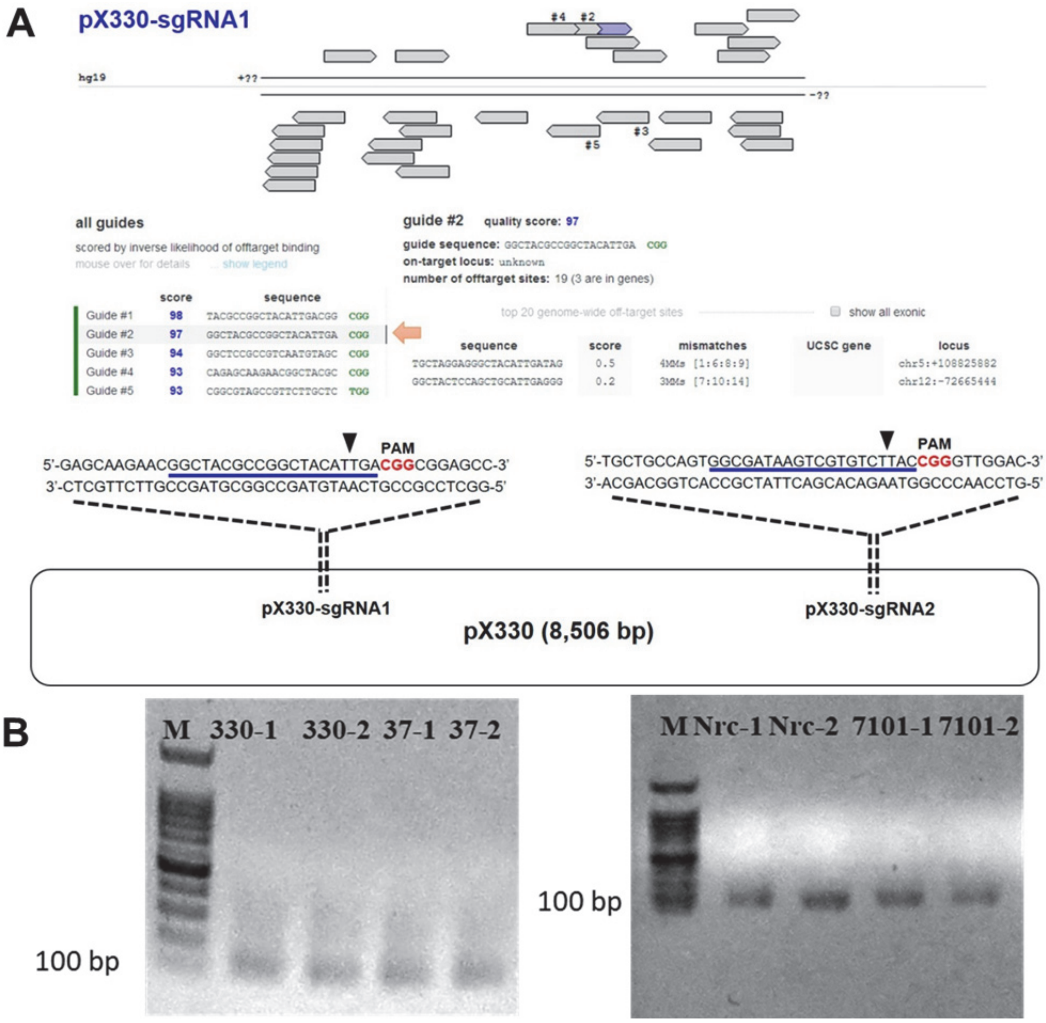

Figure S2. Design and synthesis of sgRNA. (A) Design of the CRISPR guide sequence pX330-sgRNA1. (B) Preparation of DNA template for sgRNA by overlapping PCR with three oligos, a target-specific oligo (X-sgRNA-P) containing the T7 promoter and target sequence, and two universal oligos (sgRNA-F and sgRNA-R) of an sgRNA constant sequence contained in the crRNA-tracrRNA chimera.

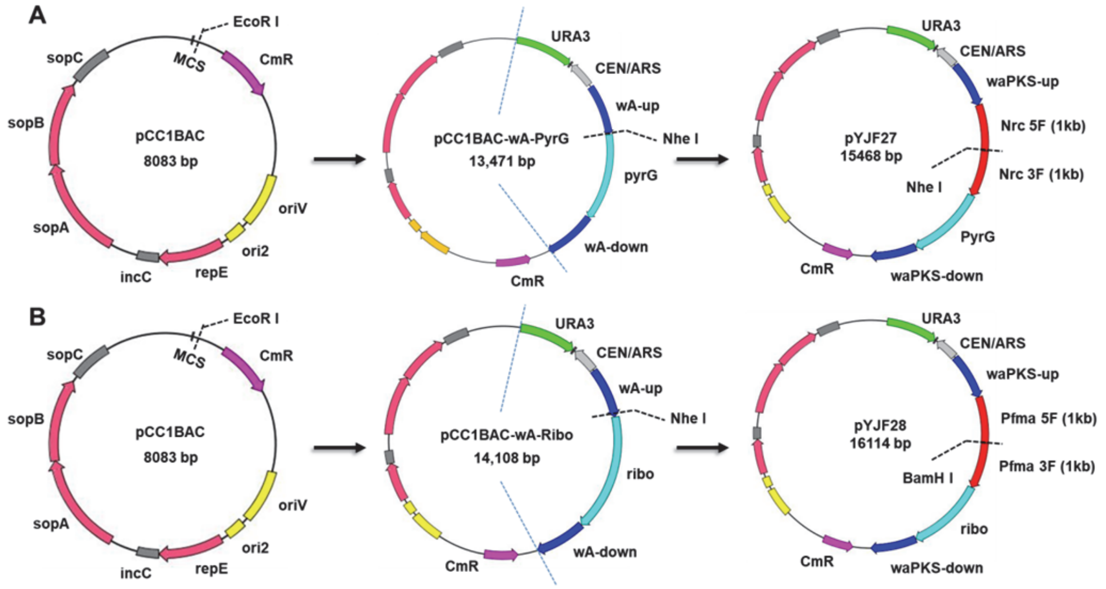

Figure S3. Construction of shuttle pCC1BAC vectors. (A) Construction of pYJF27 (*pCC1BAC-wA-pyrG-Nrc)*. Auxotrophy marker *PyrG* was introduced into pCC1BAC, *Nhe I* was designed to be restriction site (pYJF24), and 5'-flank (5F) and 3'-flank (3F) of *Nrc* gene clusters were cloned into pCC1BAC. (B) Construction of pYJF28 (*pCC1BAC-wA-ribo-Pfma*). Auxotrophy marker *Ribo* was constructed to pCC1BAC, *Nhe I* was designed to be restriction site (pYJF26), and 5'-flank (5F) and 3'-flank (3F) of *Pfma* gene clusters were cloned into pCC1BAC.

Supplemental Table

Table S1. Primers used in this study.

Primers	Oligonucleotide sequence (5’-3’)	Uses
Cas9-pET28a-F1	CGCGGATCCGAATTCGAGCTCATGG ACAAGAAGTACAGCAT	Create the vector pYJF22
Cas9-pET28a-R1	GGCCATTTCGATCACGATGTTCTCG GGC	Create the vector pYJF22
Cas9-pET28a-F2	GCCGGAAGCTGATCAACGGCATCC GGG	Create the vector pYJF22
Cas9-pET28a-R2	CGAGTGCGGCCGCAAGCTTGTCGA CGTCGCCTCCCAGCTGAGACAGGT CG	Create the vector pYJF22
sgRNA-F	GTTTTAGAGCTAGAAATAGCAAGTT AAAATAAGGCTAGTC	Intermediate primers for preparing the *in vitro* sgRNA template
sgRNA-R	AAAAGCACCGACTCGGTGCCACTT TTTCAAGTTGATAACGGACTAGCCT TATTTTAACT	End primers for preparing the *in vitro* sgRNA template
X330-sgR1-P	TAATACGACTCACTATAGGCTACGC CGGCTACATTGAGTTTTAGAGCTAG AAATAGCAA	Primer for sgRNA cleaved to pX330
X330-sgR2-P	TAATACGACTCACTATAGGCGATAA	Primer for sgRNA cleaved

	GTCGTGTCTTACGTTTTAGAGCTAGAAATAGCAA	to pX330
PZ37-sgR1-P	TAATACGACTCACTATAGGATTTGATTGCGACGCTCCGTTTTAGAGCTAGAAATAGCAA	Primer for sgRNA cleaved to pYPZ37
PZ37-sgR2-P	TAATACGACTCACTATAGGCGATTCTTGCGGATCTCGGTTTTAGAGCTAGAAATAGCAA	Primer for sgRNA cleaved to pYPZ37
BAC-F1	GAATTGTAATACGACTCACTATAGGGCCATTATACGAAGTTATGCACCACGC	Construction of shuttle pCC1BAC vectors
BAC-R1	GGTGAGACTATAGAATACTCAAGCTCTGCTGTCAGTACGCGAAGATCTCC	Construction of shuttle pCC1BAC vectors
wAup-Nrc-F	GGACTTGACTCTCCTTCTCCTGATCAGTGGGCGTTCTTCTCAAATTGCGG	Construction of shuttle pCC1BAC vectors
wAup-Nrc-R	GCACATGTGGTCGACCGAGTGTTTAAACTTCTACGTCGCGACGAACAAC	Construction of shuttle pCC1BAC vectors
Nrc-PyrG-F	GTTGTTCGTCGCGACGTAGAAGTTTAAACACTCGGTCGACCACATGTGC	Construction of shuttle pCC1BAC vector pYJF27
Nrc-PyrG-R	CAGACACAGAATAACTCTCGCTAGCGATGGGGCTGTTTCACATTTGCCTC	Construction of shuttle pCC1BAC vector pYJF27

riboWA-F	GACTTGACTCTCCTTCTCCTGATCGGATCCCACATGGGATTAAAATATGGTG	Construction of shuttle pCC1BAC vector pYJF28
riboWA-R	TAGTAAAGTGATTCGCGTCATGCGGCCGCGACCGGGGTCTCTAGTTTTCC	Construction of shuttle pCC1BAC vector pYJF28
Nrc-5F-F	GCGGACTTGACTCTCCTTCTCCTGATCGGCAGGTAGTGGAGAGGTTTAAGCAG	Upstream primer for 5F of *Nrc* gene cluster
Nrc-5F-R	GCAGCGAGCTCTCGACGGTGGC	Downstream primer for 5F of *Nrc* gene cluster
Nrc-3F-F	GCATAAGGAGATGCGTGTGCAAGGG	Upstream primer for 3F of *Nrc* gene cluster
Nrc-3F-R	CATATTTCGTCAGACACAGAATAACTCTCGTGAGGGTTGGGTTCGATTTTTGCGG	Downstream primer for 3F of *Nrc* gene cluster
7101-5F-F	GCGGACTTGACTCTCCTTCTCCTGATCGTAAGGTACAGGTAACCGGTCAGGGC	Upstream primer for 5F of *Pfma* gene cluster
7101-5F-R	CGTGATTTCATGGTGATTTTGGG	Downstream primer for 5F of *Pfma* gene cluster
7101-3F-F	GGGCCGGCGCCCGCAACCGGTGCC	Upstream primer for 3F of *Pfma* gene cluster

7101-3F-R	CAACACCATATTTTAATCCCATGTGATCTGGTATATGGTAATATGATTATG	Downstream primer for 3F of *Pfma* gene cluster
Nrc-sgR1-P	TAATACGACTCACTATAGGAAACGGGGAGTTATCGGCGTTTTAGAGCTAGAAATAGCAA	Primer for sgRNA cleaved to *Nrc* gene cluster
Nrc-sgR2-P	TAATACGACTCACTATAGGCACCGCTGCGAGTTCGGTGGTTTTAGAGCTAGAAATAGCAA	Primer for sgRNA cleaved to *Nrc* gene cluster
7101-sgR1-P	TAATACGACTCACTATAGGATTCCGAGCTCGCGGGTAGTTTTAGAGCTAGAAATAGCAA	Primer for sgRNA cleaved to *Pfma* gene cluster
7101-sgR2-P	TAATACGACTCACTATAGGTGTGAGCCCTTCGCTATCGTTTTAGAGCTAGAAATAGCAA	Primer for sgRNA cleaved to *Pfma* gene cluster
Nrc-F5	GAGGTCGATGGACATCATGCGC	Validation primer for *Nrc* gene cluster
Nrc-R5	GGAGAAGCCGTCACAAACGTCTCC	Validation primer for *Nrc* gene cluster
7101-F5	CCACTGTTCCGGCGTATGCCAACTG	Validation primer for *Pfma* gene cluster
7101-R5	CCCAGACTTGGCCAGGTTCAGCTATG	Validation primer for *Pfma* gene cluster
